# Ginseng: a promising neuroprotective strategy in stroke

**DOI:** 10.3389/fncel.2014.00457

**Published:** 2015-01-20

**Authors:** Vaibhav Rastogi, Juan Santiago-Moreno, Sylvain Doré

**Affiliations:** ^1^Departments of Anesthesiology, Center for Translational Research in Neurodegenerative Disease, University of Florida College of MedicineGainesville, FL, USA; ^2^Departments of Neurology, Center for Translational Research in Neurodegenerative Disease, University of Florida College of MedicineGainesville, FL, USA; ^3^Departments of Psychiatry, Center for Translational Research in Neurodegenerative Disease, University of Florida College of MedicineGainesville, FL, USA; ^4^Departments of Neuroscience, Center for Translational Research in Neurodegenerative Disease, University of Florida College of MedicineGainesville, FL, USA

**Keywords:** ginseng, stroke, neuroprotection, ginsenosides, ischemia, hemorrhage

## Abstract

Ginseng is one of the most widely used herbal medicines in the world. It has been used in the treatment of various ailments and to boost immunity for centuries; especially in Asian countries. The most common ginseng variant in traditional herbal medicine is ginseng, which is made from the peeled and dried root of *Panax Ginseng*. Ginseng has been suggested as an effective treatment for a vast array of neurological disorders, including stroke and other acute and chronic neurodegenerative disorders. Ginseng’s neuroprotective effects are focused on the maintenance of homeostasis. This review involves a comprehensive literature search that highlights aspects of ginseng’s putative neuroprotective effectiveness, focusing on stroke. Attenuation of inflammation through inhibition of various proinflammatory mediators, along with suppression of oxidative stress by various mechanisms, including activation of the cytoprotective transcriptional factor Nrf2, which results in decrease in reactive oxygen species, could account for its neuroprotective efficacy. It can also prevent neuronal death as a result of stroke, thus decreasing anatomical and functional stroke damage. Although there are diverse studies that have investigated the mechanisms involved in the efficacy of ginseng in treating disorders, there is still much that needs to be clarified. Both *in vitro* and *in vivo* studies including randomized controlled clinical trials are necessary to develop in-depth knowledge of ginseng and its practical applications.

## Introduction

Ginseng is a broad term that refers to a group of 11 species of perennial plants belonging to the *Panax* genus under the family *Araliaceae*. The commercially available herbal formulations of ginseng are extracted from the root of these plants. It has been used for more than 2000 years mainly in China, Korea and Japan. The most commonly used herbal derivative of ginseng is Korean ginseng which is derived from the peeled, steamed, and dried root of *Panax ginseng* also commonly known as Korean ginseng. Although there are other variants of ginseng that contain many of the same compounds and medicinal properties, we will mainly focus on *Panax ginseng* and its constituents. The chemical constituents of ginseng include triterpene saponins, polysaccharides, peptidoglycans, nitrogen-containing compounds, fatty acids, carbohydrates and phenolic compounds (Sticher, 1998). It also contains essential oil-containing polyacetylenes and sesquiterpenes (Sticher, 1998). Ginsenosides are the major active components in ginseng; they are a form of triterpene glycosides (saponins). Of the 150 ginsenosides that have been isolated from ginseng, 40 have been found in *Panax ginseng* alone (Christensen, 2009). These mainly include Rb1, Rb2, Rc, Rd, Rg1, Rg2, Rh1, and Re (Attele et al., 1999).

The Greek word “panax” means “cure-all” and true to its name, ginseng has proven to have a wide variety of medicinal uses. Ginseng can improve pulmonary lung function in stable chronic obstructive pulmonary disorder patients (An et al., 2011). Korean ginseng has been shown to provide symptomatic relief in the patients with complaints of erectile dysfunction (Hong et al., 2002). A large number of carcinomas, including those associated with smoking, could potentially be prevented by the regular use of ginseng (Yun and Choi, 1995). Ginseng’s efficacy in type-2 diabetics has been illustrated by the fact that the patients had a decrease in the fasting blood glucose, weight loss and HbA1c (glycated hemoglobin) along with improvement in mood and psychophysical performance (Sotaniemi et al., 1995). Ginseng also possesses numerous cardiovascular benefits that are mainly due to its cardioprotective and anti-hypertensive effects; it can also attenuate myocardial hypertrophy and heart failure (Karmazyn et al., 2011).

Ginseng is also known to affect various aspects of neurodevelopmental, neurodegenerative and neuropsychiatric disorders (Kim et al., 2013). Stroke is the 4th leading cause of death in United States with an estimated 1 death every 4 min. Stroke recurs in 1 out of every 4 stroke patients. Approximately 87% of strokes are a result of ischemic insult and 13% are hemorrhagic strokes. Subarachnoid hemorrhage (SAH) accounts for approximately 3% of all strokes, has an incidence rate of 30,000 cases per year (King, 1997) and is mostly seen after brain aneurysm rupture or in traumatic brain injury (TBI) patients. Stroke is also one of the leading causes of long term disability (Go et al., 2013). These are reasons for stroke’s high magnitude of mortality and morbidity. From this information, one can see that present preventive and treatment strategies are not sufficient to curb this health menace. Because of this, there is a need to look for other treatment modalities and ginseng has shown promising evidence in this regard.

## Putative ginseng neuroprotection and mechanisms of action in stroke

The role of ginseng and its saponins in stroke prevention and treatment has been an area of great interest for researchers. The studies have elucidated various mechanisms contributing to the efficacy of ginseng in ischemic stroke.

### Anti-inflammatory effects

Perhaps the most intriguing property of ginseng is its anti-inflammatory effect. Microglial activation plays an important role in inflammation as it develops many properties of macrophages. Activated microglia have both pro- and anti-inflammatory properties (Iadecola and Anrather, 2011). It has two activated phenotypes: M1, which is pro-inflammatory and is activated by inciting factors such as stress, inflammatory challenge, and M2, which is anti-inflammatory and is activated by anti-inflammatory cytokines such as interleukin 4 (IL-4; Rojo et al., 2014). The M2 phenotype plays a role in post-ischemic inflammation resolution and tissue repair through secreting mediators such as transforming growth factor-β (TGF-β) and IL-10 (Denes et al., 2007). The pro-inflammatory role of M1 is mediated by various inflammatory mediators including reactive oxygen intermediates, nitric oxide (NO), proteases (Colton and Gilbert, 1987; Banati et al., 1993a,b), and cytokines including IL-1, IL-6, and interferon-γ (IFN-γ; Sawada et al., 1989; Dickson et al., 1993; Kleinig and Vink, 2009). Ginsenoside Rb1 has been shown to repress microglial M1 activation and decrease the pro-inflammatory cytokines IL-6 in a transient middle cerebral artery occlusion (MCAO) rat model. Zhu et al. found a decrease in the total infarct volume and modified Neurological Severity Score (mNSS) on intranasal administration of 12.5 mg/kg ginsenoside Rb1 (Zhu et al., 2012). Ginsenoside Rd and compound K (a metabolite of ginseng saponins) also decrease microglial M1 activation (Ye et al., 2011c; Park et al., 2012). Ye et al. demonstrated a decrease in infarct volume and mNSS on intraperitoneal (IP) administration of 50 mg/kg ginsenoside Rd in the transient MCAO rat model (Ye et al., 2011c). Park et al. also showed that IP administration of 30 mg/kg compound K to the transient MCAO mice model can decrease the total infarct volume (Park et al., 2012).

Nuclear factor-κB (NF-κB) is a transcriptional pathway involved in the regulation of inflammation through the expression of genes such as cycloygenase-2 (COX-2), inducible nitric oxide synthase (iNOS), IL-6 (Baeuerle and Henkel, 1994). Ginsenoside Rb1 suppresses total as well as phosphorylated NF-κB and inhibits its DNA binding activity which in turn suppresses neuronal death (Schneider et al., 1999) as well as decreases IL-6 levels (Wang et al., 2007) in the brain (Zhu et al., 2012). Compound K can also suppress the NF-κB pathway in mice (Park et al., 2012). Lee et al. injected healthy mice with a single dose of IP 3 mg/kg lipopolysaccharide to activate microglia and subsequently inflammation. In the treatment group ginsenoside Rb1 was administered orally at a dose of 10 or 20 mg/kg 1 h prior to lipopolysaccharide injection. Similar to the other studies they found that ginsenoside Rb1 prevents this microglial activation and subsequent release of pro-inflammatory cytokines such as IL-1β, IL-6, and pro-inflammatory COX-2 enzyme at a dosage of 20 mg/kg (Lee et al., 2013). COX-2 overexpression has also been linked to neuronal death post-ischemia in the ischemic areas as well as in the surrounding areas through an increase in the synthesis of prostaglandins (Nogawa et al., 1997; Sairanen et al., 1998; Doré et al., 2003). Ginsenoside Rd decreases the activity of COX-2 by directly decreasing COX-2 levels, so it can prevent ischemic death of neurons (Ye et al., 2011c). Like COX-2, iNOS also catalyzes the formation of the inflammatory mediator NO and its expression has been thought to be mainly in the cells involved in the inflammation such as microglia, astrocytes (Wang et al., 2007). Ginsenoside Rd has been shown to inhibit iNOS, thus decreasing NO production which decreases inflammation post-ischemia (Ye et al., 2011c).

Mitogen-activated protein kinase (MAPK) and the proline-directed kinases are another group of enzymes that enhance the formation of pro-inflammatory proteins along with other functions (Kyriakis and Avruch, 2001). Compound K, a metabolite of ginseng inhibits phosphorylation of MAPKs in mice, thus inhibiting their activation; this translates into decreased production of pro-inflammatory proteins (Park et al., 2012). The p38 MAPK can cause down-regulation of heme oxygenase 1 (HO-1) expression, which is a rate-controlling enzyme for heme metabolization (Tenhunen et al., 1968; Naidu et al., 2009). HO-1 in itself has potent anti-inflammatory, antioxidant and anti-apoptotic properties (Naidu et al., 2009; Gozzelino et al., 2010). HO-1 can suppress NF-κB, and consequently it can inhibit IL-1β expression which is pro-inflammatory (Rushworth et al., 2008). Compound K treated mice showed an enhancement in HO-1 expression for resolution of inflammation in activated microglia (Park et al., 2012). Lee et al. used Korean ginseng instead of individual components such as ginsenoside Rd and ginsenoside Rb1; they also showed that it possesses anti-inflammatory properties similar to its components when administered at 100 mg/kg orally to transient MCAO rat models (Lee et al., 2011).

One of the major causes of morbidity and mortality post-stroke is cerebral edema. Blood brain barrier disruption plays a major role in the causation of the edema. Ginsenoside Rg1 prevents the disruption of this barrier by inhibiting the expression of aquaporin 4. When IP ginsenoside Rg1 treatment at a dosage of 20 mg/kg was given in combination with acetazolamide (an aquaporin 4 reversible inhibitor) (Tanimura et al., 2009), neurological recovery was greater than when administered alone to the transient MCAO rat models (Zhou et al., 2014). Astrocytes are the most abundant glial cells; they have neuronal supportive effects during normal conditions but during ischemia or injury they act pro-inflammatory and cause tissue scar formation (Hayakawa et al., 2010). Yoshikawa et al. showed that 300 μg/kg intravenous (IV) ginsenoside Rb1 decreases cerebral edema and inhibits delayed neuronal death post-ischemic insult through the inhibition of astrocyte activity in the peri-infarct region in thromboembolic stroke in monkeys (Yoshikawa et al., 2008).

### Anti-apoptotic effects

Another important role of ginseng is the inhibition of apoptosis or delayed cell death. Bcl-2 is the major anti-apoptotic protein which is located mainly on the outer membrane of mitochondria; (Monaghan et al., 1992; Reed, 1997) this delineates the importance of mitochondria in apoptosis. Bcl-2-associated X protein (BAX) is the major pro-apoptotic protein that homodimerizes and heterodimerizes with Bcl-2, such that a reciprocity exists between both BAX and Bcl-2 (Oltvai et al., 1993). The dynamic balance between Bcl-2 and BAX plays a major role in regulating apoptosis. Ginsenoside Rb1 when administered at a dosage of 30 mg/kg IP to transient MCAO rat models has been shown to increase Bcl-2 protein and decrease BAX protein in rats (Yuan et al., 2007). Zhang et al. also showed that ginsenoside Rg2 increased Bcl-2 protein and decrease BAX protein when given to transient MCAO rat models at a dose of 2.5, 5 and 10 mg/kg IV (Zhang et al., 2008).

Cytochrome c, a small heme protein mainly located on inner membrane of mitochondria, is linked to the respiratory chain complex. It also plays an important role in apoptosis (Gonzales and Neupert, 1990). Apoptotic cells show an increase in cytosolic cytochrome c with a corresponding decrease in mitochondrial one, Bcl-2 overexpression can prevent this efflux to the cytosol, thus preventing apoptosis (Yang et al., 1997). Liang et al. used human neuroblastoma SH-SY5Y cells and exposed them to oxygen-glucose deprivation. They showed that ginsenoside Rb1 at 1 and 10 μmol/l concentration inhibits apoptosis by impeding cytochrome c and apoptosis-inducing factor (AIF) release from mitochondria (Liang et al., 2013). Apoptotic stimuli induce cytochrome c production which causes conversion of pro caspase-3 into active caspase-3, thus causing cell death (Reed, 1997). Caspase 3 is a protease involved in the cleavage of substrates that results in apoptosis (Nicholson and Thornberry, 1997); it can also potentiate apoptosis by causing release of cytochrome c as well as by conversion of anti-apoptotic Bcl-2 into pro-apoptotic by cleaving its carboxy-terminal (Cheng et al., 1997). Ginsenoside Rd’s anti-apoptotic properties are evident by the fact that it attenuates mitochondrial release of AIF, caspase 3 and cytochrome c during transient MCAO in rats when given in dose of 50 mg/kg IP, thus decreasing the total infarct volume (Ye et al., 2011d). Ginsenoside Rb1 also decreases the activity of caspase-3 as demonstrated by Gao et al. when they administered 40 mg/kg ginsenoside Rb1 IV to transient MCAO rat models (Gao et al., 2010), thus inhibiting cell death post-ischemia.

Various other proteins inhibit apoptosis such as neuronal AIF, which inactivates caspase-3 and caspase-7, ginsenoside Rb1 increases the expression of neuronal apoptosis inhibitory protein in rats (Yuan et al., 2007). Heat shock protein 70 is another apoptosis inhibitory protein that has been shown to prolong survival post-ischemic insult through a decrease in cytochrome c release, which subsequently inhibits the down-stream apoptotic pathway (Tsuchiya et al., 2003). Ginsenoside Rg2-treated rats showed an increase in heat shock protein 70 expression post-ischemia which underlines its anti-apoptotic role (Zhang et al., 2008). Other examples include tumor suppressor protein p53, which has been associated with apoptosis in response to brain ischemia (Morrison et al., 1996) or injury, probably through BAX activation (Miyashita and Reed, 1995). The p53 expression was decreased in rats after ginsenoside Rg2 administration at various doses, which might be another reason for its protective effect (Zhang et al., 2008). Thus, ginsenoside Rg2 can potentially prevent vascular dementia which is a long term sequel of ischemic stroke, by inhibiting neuronal apoptosis post-acute ischemic insult (Zhang et al., 2008). Neurotrophic factors are some proteins required for cell survival, growth, and maintenance; ginsenoside Rb1 also increases the expression and regulation of brain-derived neurotrophic factor (BDNF; Gao et al., 2010), and glial-derived neurotrophic factors (GDNF) during transient MCAO in rats (Yuan et al., 2007).

### Anti-oxidative effects

Attenuation of oxidative stress might be another explanation for ginseng’s neuroprotective effects as described above. Oxidative stress is the accumulation of reactive oxygen species (ROS) due to its decreased degradation; these ROS can damage lipids, DNA, RNA and proteins leading to neuronal dysfunction and cell death. ROS can also cause the peroxidation of lipids because lipids are present in biomembranes, thus damaging neuronal structure. Lipid peroxides can be reduced by glutathione peroxidase, thus protecting the neurons. ROS are released in excessive amounts from the microglia M1 phenotype. ROS in turn cause the activation of the transcriptional factor Nrf2 [originally named: nuclear factor (erythroid-derived 2)-like 2], which plays a vital role in various cell properties and notably bringing back the microglia to the steady state (Rojo et al., 2014).

**Figure 1:**
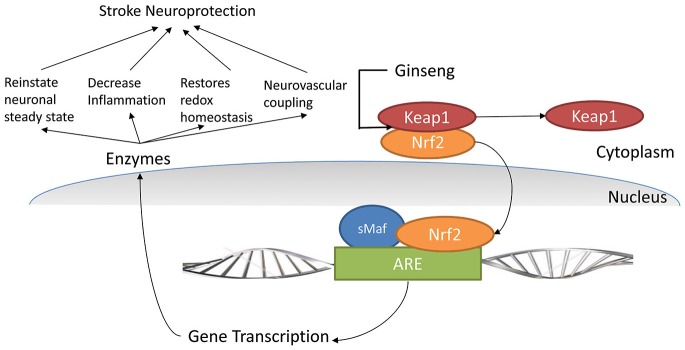
**Nrf2 pathway and its role in cellular protection**. (Nrf2: nuclear factor (erythroid-derived 2)-like 2, ARE: Antioxidant response element, Keap1: Kelch-like ECH-associated protein 1, sMaf: Small Maf proteins, CREB: cyclicAMP response element-binding protein, ATF - 4: activating transcription factor 4 protein).

Nrf2 is the major transcription factor that binds to the antioxidant response element (ARE), which in itself is a multiple gene regulator, also including genes for phase 2 enzymes (Chen and Kunsch, 2004; Leonardo and Doré, 2011). In normal body dynamics, Nrf2 is bound to kelch-like ECH-associated protein 1 (Keap1) and is inactivated. During stress, Keap1 disseminates from Nrf2 (Itoh et al., 1999) and translocates in the nucleus and thus Nrf2 causes initiation of ARE pathway mediated cytoprotection (Wang et al., 2013; Figure 1). This activated Nrf2 restores microglia’s steady state by regulation of ARE-associated genes such as enzymes involved in the degradation of ROS, including SOD, glutathione peroxidase and enzymes involved in synthesis of reducing factors such as glucose-6 phosphate dehydrogenase [involved in synthesis of reduced nicotinamide adenine dinucleotide phosphate (NADPH)]. Other genes include enzymes involved in regeneration of reduced cofactors such as glutathione reductase and enzymes involved in reduction of oxidized cofactors such as thioredoxin reductase. These also include genes for ferritin required for clearance of metals as well as redox transporters such as cysteine-glutamine transporter and enzymes involved in antioxidant production such as HO-1 (Rojo et al., 2014). Triterpenoids can activate Nrf2 by dissociating Keap1 from Nrf2 (Figure 1; Liby et al., 2005). Ginseng active constituents include triterpenes as previously described; thus ginseng can also upregulate ARE pathway-mediated neuroprotection. Hwang and Jeong (2010) previously suggested that Rb1 would be protective in SH-SY5Y cells via an ER-dependent Gbeta1/PI3K/Akt-Nrf2 signaling pathway. And similarly, Du et al. (2013) proposed that the potential beneficial effect of Ginseng Rb1 would be via Akt/Nrf2 in the human SK-N-SH dopaminergic cell line.

Ginsenoside Re, a ginseng saponin can increase the activity of SOD and glutathione peroxidase, and subsequently alleviate oxidative stress when administered to transient MCAO rat models as described by Zhou et al. (2006). It also decreases the amount of malondialdehyde, which is an oxidative stress marker. Ginsenoside Rd, similar to ginsenoside Rb1, can alleviate oxidative stress induced damage to DNA, RNA, proteins, and lipids, and it also preserves endogenous antioxidant activities *in vivo*. They found that on administering 50 mg/kg IP ginsenoside Rd to transient MCAO mice and rat models respectively, there was a decrease in the infarct volumes and an improvement in behavioral tests (Ye et al., 2011b,c). Ye et al. also used an *in vitro* mitochondrial model (intact nonsynaptosomal mitochondria obtained from rats) to show that ginsenoside Rd protects mitochondria from calcium-induced mitochondrial swelling or damage by a decrease in production of ROS (Ye et al., 2011d). Compound K also attenuates oxidative stress in post-ischemic rats by decreasing ROS production by microglia by attenuating NADPH oxidase, which is inhibited by carbon monoxide, a product of HO-1 activity (Nakahira et al., 2006; Park et al., 2012). Kim et al. administered ethanolic *Panax Ginseng* extracts IP at a dose of 200 mg/kg to transient forebrain ischemia rat models whereas Ban et al. administered 100 mg/kg Korean red ginseng orally to transient MCAO rat models (Kim et al., 2009; Table 1).

**Table 1:** **Anatomical and behavioral effects of various ginseng extracts on *in vivo* cerebral ischemia models**.

Model	Ginsenoside	Species	Dosage and route	Anatomical outcome	Assessment time after surgery	Behavioral outcome	Reference
MCAO (2h)	Rb1	Rats	40 mg/kg IV		3 and 12h, 1, 2, 3, 5 and 10 days	Decrease mNSS	Gao et al. (2010)
MCAO (2h)	KRG	Rats	100 mg/kg/day PO		1, 3, and 7 days	Decrease mNSS, corner test	Ban et al. (2012)
MCAO (permanent)	dgRb1 (Dihydroginsenoside Rb1)	Stroke-prone spontaneously hypertensive rats (SHR-SP)	Immediately post-MCAO IV bolus of 60 µl (0.6 or 6 µg/60 µl) followed by maintenance IV dose of (0.6 or 6 µg/day)	Total infarct volume decrease	2 and 4 weeks	Decrease Morris water maze test	Sakanaka et al. (2007)
MCAO (2h)	Rh2	Rats	Rh2 and acid treated ginseng PO	Total infarct volume decrease			Park et al. (2004)
MCAO (2h)	Fermented ginseng	Rats	Fermented ginseng and ginseng PO	Total infarct volume decrease			Bae et al. (2004)
MCAO (30 min)	Compound K	Mice	30 mg/kg IP	Total infarct volume decrease			Park et al. (2012)
MCAO (permanent)	Rb1	SHR-SP Rats	Immediately post-MCAO IV bolus of (6 or 60 µg/60 µL) followed by maintenance IV dose of (6 or 60 µg/day)	Total infarct volume decrease. Prevention of cortical infarction and secondary thalamic degeneration	2 and 4 weeks	Decrease Morris water maze test	Zhang et al. (2006)
MCAO (permanent)	KRG	SHR-SP Rats	0.006–6.0 µg/d IV	Total infarct volume decrease	2 and 4 weeks	Decrease Morris water maze test	Zhang et al. (1998)
MCAO (2h)	KRG	Rats	100 mg/kg/day PO	Total infarct volume decrease	1, 3, and 7 days	Decrease mNSS, corner turn test	Lee et al. (2011)
MCAO (90 min)	Rb1	Rats	12.5 mg/kg IN or IV	Total infarct volume decrease			Lu et al. (2011)
MCAO (2h)	Rb1	Rats	12.5 mg/kg IN	Total infarct volume decrease	6, 24 and 72h	Decrease mNSS	Zhu et al. (2012)
MCAO (permanent)	Rb1, Rg1	Rats	Rb1 10, 20, 40 mg/kg IV	Rb1 decrease infarct size	24 h	Decrease Rb1 NDS,	Zhang and Liu (1996)
MCAO (2h)			Rg1 40 mg/kg IV	Rg1 ineffective		Ineffective Rg1 NDS
MCAO (2h)	Black ginseng	Rats	100, 400 mg/kg PO		2 weeks	Decrease Morris water maze test	Park et al. (2011)
MCAO (permanent)	Ginseng total saponins	Rats	25 mg/kg IP		1, 3, 7 and 14 days	Decrease NDS	Zheng et al. (2011)
MCAO (2h) MCAO (permanent)	Rd	Rats	Dose dependent study 1–50 mg/kg IV	Infarct size decrease from 10 mg/kg to 50 mg/kg at different time-points. Infarct size decrease with Rd at 2h (36–44%) or 4 h (31–40%), but no change at or after 8 h in MCAO (2h). Infarct size decrease after permanent MCAO	1, 3, 7, 14, 21, 28, and 42 days	Dose dependent improvements in mNSS, modified sticky tape test, corner test	Ye et al. (2011a)
MCAO (1h)	Rd	Mice	0.1–200 mg/kg IP in dose response study 50 mg/kg IP in therapeutic window study	Infarct volume decrease with greatest at 50 mg/kg while the 0.1, 1 and 200 mg/kg were ineffective. Infarct volume decrease with Rd at 2h (36.3%) or 4 h (34.6%) but no change at 8 h.	1 and 14 days	1)Decrease in a Battery of 2 tests Postural reflex test, Forelimb placing test borderline (Belayev et al., 1996); 2) Increase in a Battery of 6 tests (Garcia et al., 1995), increase.	Ye et al. (2011b)
MCAO (2h)	Rd	Rats	0.1–200 mg/kg IP	Infarct volume decrease greatest at 50 mg/kg at day 1 while the 0.1, 1 and 200 mg/kg doses were ineffective	1, 3, 7, 14, 21, 28, and 42 days	Decrease mNSS	Ye et al. (2011c)
MCAO (2h)	Rd	Rats	50 mg/kg IP	Total infarct volume decrease	1 and 14 days	1) Decrease in a Battery of 2 tests (Belayev et al., 1996), 2) Increase in a Battery of 6 tests (Garcia et al., 1995)	Ye et al. (2011d)
MCAO (2h)	Rg1	Rats	20 mg/kg IP		6 h, 1, 3, 7 and 14 days	Decrease NDS	Zhou et al. (2014)
MCAO (2h)	Rb1	Rats	20, 40, 80 mg/kg	Total infarct volume decrease		Decrease NDS	Liu et al. (2013)
MCAO (1h)	Ginseng	Mice	360 mg/kg	Total infarct volume decrease		Better Rotarod scores	Cheon et al. (2013)
MCAO (2h)	Rd	Rats	30 mg/kg IP 1 h before MCAO, 10 mg/kg OD until sacrifice	Total infarct volume decrease, Reduction in hippocampal tissue loss	Post- operative days 26–32	Improved performance in Morris Water Maze and Novel Object Recognition Test	Zhang et al. (2014)
BCCAO (3 min or 3.5 min)	Rb1	Gerbils	Immediately post-BCCAO, IV bolus of 2 µL (2.5 or 25 ng/2 µL) followed by maintenance IV (60 or 600 ng/day) IP		7 day	Step-down Passive avoidance task method, Increase	Lim et al. (1997)
BCCAO (5 min)	Rb1, Rg1, Ro	Gerbils	Ginseng powder (RGP) (0.6, 0.9 or 1.5 g/kg PO Crude ginseng saponin (CGS) and Crude ginseng no-saponin (CGNS) (both 50 or 100 mg/kg) IP Rb1, Rg1, Ro (10 or 20 mg/kg for each) IP		7 day	Passive avoidance task. RGP, CGS, CGNS (100 mg/kg), Rb1 preischemic dose increase. Postischemic RGP, CGS or Rb1 was ineffective	Wen et al. (1996)
Thromboembolic stroke of left MCA	Rb1	Monkeys	300 µg/kg IV	Brain edema decrease	1, 6, and 24 h and 2, 4, and 7 days	NDS decrease	Yoshikawa et al. (2008)

*IN: intranasal; IP: Intraperitoneal, IV: Intravenous, mNSS: modified Neurological Severity Score, NDS: Neurological Deficit Score, NIHSS: National Institutes of Health Stroke Scale, OD: Once Daily, PO: Per oral*.

### Ginseng potential other beneficial effects

Additionally, ginsenosides can attenuate autophagy, a process in which cells degrade dysfunctional cellular components to survive stressful conditions, the deregulation of which can be seen in disease conditions. Ischemic neuronal death is associated with an increase in autophagy via the formation of autophagosomes (Wen et al., 2008). Lu et al. demonstrated that ginsenoside Rb1 can decrease autophagy via a decrease in the proteins associated with it such as microtubule-associated protein 1A light chain 3 (LC3), Beclin 1 (Lu et al., 2011). An intranasal administration of 12.5 mg/kg ginsenoside Rb1 to transient MCAO rat models was used that also exerted significant brain targeting effects due to direct passage of ginsenoside Rb1 to the brain from the nose through the olfactory epithelium (Lu et al., 2011).

Neurogenesis might also be a reasonable explanation. In the event of ischemic insult to the brain, neuronal precursors migrate to that area, to replace the damaged neurons (Parent et al., 2002). Ginsenoside Rb1 increases the numbers of these neuronal precursors, which can cause recovery of neural behavior post-ischemia (Gao et al., 2010). Zheng et al. suggested a role for ginseng total saponins in the proliferation of endogenous neural stem cells which can help in neuronal regeneration that can overcome neuronal loss induced deficits seen due to permanent ischemia in rats. They gave IP ginseng total saponins to rats at a dosage of 25 mg/kg and observed a decrease in the Neurological Deficit Score (NDS; Zheng et al., 2011). Such a hypothesis has attracted much attention; although, one should remain realistic about the hope of reestablishing all neuronal connections in an adult brain.

Preservation of energy metabolism in the brain post-stroke is a possible pathway for neuroprotective effects of ginseng in the brain. Ye et al. showed that ginsenoside Rd post-ischemia can preserve the mitochondrial electron transport chain in mice brain (ischemic cortex and striatum), and thus sustain energy generation (Ye et al., 2011b). It also significantly decreases the anaerobic glycolysis end-product, lactate, and increases aerobic glycolysis and thus its end-product, pyruvate, thus improving the energy status of the brain (Ye et al., 2011d). Ginsenoside Re has been shown to improve the fluidity of the mitochondrial membrane by reducing its microviscosity because fluidity of the mitochondrial membrane is important for energy generation (Zhou et al., 2006).

**Figure 2:**
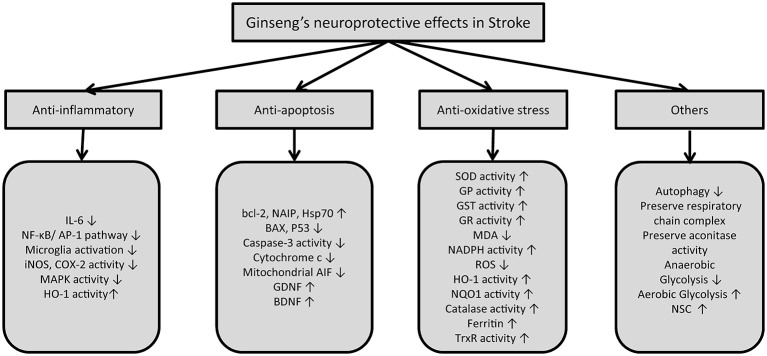
**Summary of mechanisms of action of ginseng and its constituents in stroke**. (IL-6: Interleukin-6, NF-κB: Nuclear factor-κB, AP-1: Activator protein-1, iNOS: Inducible nitric oxide synthase, COX-2: Cycloxygenase-2, MAPK: Mitogen-activated protein kinase, HO-1: Heme oxygenase-1, AIF: apoptosis inducing factor, GDNF: Glial-derived neurotrophic factors, BDNF: Brain-derived neurotrophic factor, SOD: Superoxide dismutase, GP: Glutathione peroxidase, GST: Glutathione S-transferase, GR: Glutathione reductase, MDA: Malondialdehyde, NADPH: Nicotinamide adenine dinucleotide phosphate-diaphorase, NQO1: NADPH quinone reductase 1, ROS: Reactive oxygen species, TrxR: Thioredoxin reductase).

Black ginseng has been suggested to help prevent vascular dementia potentially by increasing hippocampal choline acetyltransferase activity, which increases acetylcholine levels. Park et al. administered 100, 400 mg/kg black ginseng orally to transient MCAO rat models and noticed an improvement in the Morris water maze test. To get black ginseng, the roots are steamed and dried a number of times, which has been suggested to increase the level of saponins (Park et al., 2011). Endothelial dysfunction is one of the underlying pathophysiologies of the vascular diseases such as stroke and myocardial infarction; it is basically the disruption of endothelial homeostasis, making it pro-inflammatory and pro-thrombotic, which causes a reduction in vasodilation (Endemann and Schiffrin, 2004). Endothelium-dependent vasodilation can be impaired by various factors, including elevated levels of homocysteine in the blood that impairs the endothelium by producing oxidative stress in normotensive subjects as well as hypertensive patients (Virdis et al., 2001). Ginsenoside Rb1 has been shown to decrease homocysteine-induced, endothelium-dependent vasodilatation when administered to coronary arteries harvested from pig’s heart at a dosage of 10 μM (Zhou et al., 2005), and inhibition of endothelial proliferation when administered to a mouse lymph node endothelial cell line (SVEC4-10) and human umbilical vein endothelial cells at a dose of 10 μM (Ohashi et al., 2006). Lan et al., through both *in vivo* and *in vitro* experiments using male rats and human umbilical vein endothelial cells, suggested that ginsenoside Rb1 actions might be due to a decrease in homocysteine-induced impairment of NOS, thus increasing NO, which is a vasodilator; subsequently, it can prevent homocysteine-induced endothelial dysfunction (Lan et al., 2011). Ohashi et al. showed that ginsenoside Rb1 can inhibit homocysteine-induced ROS production from endothelial cells; subsequently, it can prevent homocysteine inhibition on endothelial proliferation (Ohashi et al., 2006). Ginsenoside Rb1 can thus prevent stroke in mice. In short, ginseng’s neuroprotective effects in cerebral ischemia are attributed to its anti-inflammatory and anti-apoptotic effects, along with attenuation of oxidative stress of the neurons (Xu et al., 2014; Figure 2).

Ginsenoside Rd, due to its highly lipophilic nature, can diffuse across biological membranes, possibly also across the blood brain barrier (Ye et al., 2011d). Ginsenoside Rd efficacy was also proposed by a human randomized, double-blind, placebo controlled multicenter trial that showed ginsenoside Rd causes a reduction in the National Institutes of Health stroke scale at 15 days post-ischemic stroke, but it did not improve overall neurological functioning (Liu et al., 2009). Although the trial results are somewhat encouraging, it had several limitations such as small sample size, no post-discharge follow up, and delayed administration of drug post-stroke at 72 h (Liu et al., 2009). Furthermore, biomarkers, circulating blood levels, etc., would be helpful for further studies.

## Ginseng protection in subarachnoid hemorrhage (SAH)

Ginsenoside Rb1 treatment has also been shown to reduce post-SAH brain edema in rats (Li et al., 2011). It also caused a significant decrease in vasospasms in the basilar artery, as well as in its lumen thickness. Vasospasm is the most common complication of SAH and leads to delayed ischemic deficits (Venkatesh Aiyagari and Diringer, 2005); thus ginsenoside Rb1 would also prevent the delayed ischemic deficits. A significant improvement was also noticed in the neuronal functioning in rats post-SAH (Li et al., 2011).

## Ginseng neuroprotection in traumatic brain injury (TBI)

TBI is another acute neurological disorder and to the same extent incorporates components of both hemorrhagic and ischemic stroke. TBI and concussions are major public health problems and causes of mortality in neurological conditions, notably in young adults. Various preclinical rat studies have demonstrated the neuroprotective effects of ginseng in TBI; however, no rigorous human studies are available. Ginseng total saponins decrease post-TBI brain edema, which is partly responsible for the focal neurological deficits seen in TBI (Xia et al., 2012). It also decreases the pro-inflammatory cytokines IL-6 and IL-1β, and increases IL-10, which is anti-inflammatory (Xia et al., 2012). It has been shown to alleviate oxidative stress post-TBI by increasing SOD and iNOS activity and decreasing malondialdehyde levels in the brain (Xia et al., 2012). Its anti-apoptotic activities can be described by the finding that it reduces caspase-3 and BAX expression and increases Bcl-2 expression (Xia et al., 2012). Other researchers have also demonstrated beneficial effects of ginseng in TBI by similar mechanisms stated above (Ji et al., 2005; Kumar et al., 2014).

## Ginseng potential in various chronic neurodegenerative disorders

Multiple clinical as well as translational studies have demonstrated the effectiveness of ginseng in various neurological disorders, both acute (ischemic and hemorrhagic stroke, TBI) and chronic (Alzheimer, Huntington and Parkinson) diseases. The main emphasis here is on preclinical chronic models and most specifically on acute preclinical models, i.e., stroke.

Alzheimer disease is the most common cause of dementia in many industrialized countries (Alzheimer’s Association, 2013). In preclinical models, ginsenoside Rb1 and ginsenoside Rg1 can increase the formation of new synapses (Mook-Jung et al., 2001). It was proposed that ginsenoside Rg1 would be involved in the regulation of the proliferation of hippocampal progenitor cells and this effect may serve as one of the elementary mechanisms underlying its nootropic and anti-aging actions (Shen and Zhang, 2004). When treated with Rg1, the NO content and NOS activity in the cerebral cortex of old rats decreased (Li et al., 1997). Fang et al. showed that ginsenoside Rg1 is protective for the neurons by impairing cerebral β amyloid accumulation in Alzheimer mouse models (Fang et al., 2012). Thus, ginseng can support neuronal survival and further promote neuronal growth and differentiation, which plays an important role in the diminution of Alzheimer disease pathology. Briefly, Ginsenoside Rb1 enhances cognition through up-regulation of hippocampal neuronal genesis (Liu et al., 2011). Along with cell genesis, synaptic plasticity also plays an important role in memory (Gerrow and Triller, 2010). Synaptic plasticity is the capability of synapses to change their strength over time in response to an alteration in their activities (Hughes, 1958). The mammalian target of rapamycin (mTOR) signaling pathway upregulates synaptic plasticity-related protein synthesis in the hippocampus, and long-term ginsenoside Rg1 treatment up-regulates mTOR signaling; thus ginsenoside Rg1 was suggested to contribute to synaptic plasticity and can prevent dementia (Yang et al., 2013). Aprikyan et al. showed that aging related dementia is associated with a significant decrease in glutamate release (Aprikyan and Gekchyan, 1988). Ginsenoside Rg1 or ginsenoside Rb1 can increase glutamate release from rat cortical synaptosomes by acting as presynaptic facilitators for 4-aminopyridine evoked glutamate release (Chang et al., 2008). Although various studies have shown an ameliorating effect of ginseng on cognitive decline, a Cochrane review by Geng et al. showed no reassuring evidence to prove the beneficial effects of *Panax ginseng* on cognition or in patients with dementia (Geng et al., 2010).

Parkinson pathology is another neurodegenerative disorder that is characterized by bradykinesia, cog-wheel rigidity, and resting tremor and can lead to dementia. Its pathophysiology involves the degeneration of dopaminergic neurons in substantia nigra, leading to a decrease in dopamine, which causes the symptoms. Ginsenoside Rb1 and ginsenoside Rg1 have been shown to support energy metabolism and preserve the structural integrity of the dopaminergic neurons in Parkinson (Radad et al., 2004). Ginsenoside Rg1 can also act against MPTP-induced apoptosis by increases in Bcl-2 formation (Chen et al., 2002).

Huntington’s disease is an inherited neurodegenerative disease with symptoms ranging from choreiform movement disorder (dyskinesia) to dementia and behavioral changes. At low doses, ginsenoside Rb1, ginsenoside Rc, and ginsenoside Rg5 have been shown to attenuate neuronal apoptosis induced by glutamate in an *in vitro* Huntington Disease model (Wu et al., 2009).

## Conclusion

The role of ginseng in neuroprotection has been an interesting topic of research for a very long time. Various mechanisms involving ginseng’s neuroprotective effects in stroke have been proposed up to this point that are basically focused on maintenance of homeostasis, anti-inflammation, anti-oxidative stress and anti-apoptosis. A more detailed characterization of the cellular and molecular pathways is required. We propose that the modulation of the Nrf2 pathway may be a way by which ginseng can build cell and organ resistance to a multitude of stresses. By inducing the expression of antioxidant and phase II detoxifying genes, Nrf2 activates a wide range of cell defense processes, thereby improving on the efficiency of cells to detoxify and eliminate various harmful substances. Also, by better understanding ginseng’s mechanism of action, it would help for the optimal design of clinical research outcomes. Dose-response relationships and therapeutic windows for ginseng and its sub-components require more elaboration. The preparation of ginseng and its compounds is highly variable; standardization of the preparations is required to compare and analyze different studies, along with pharmacokinetic and pharmacodynamic studies. Most of the preclinical studies have been performed *in vitro* or *in vivo* on rats or mice. Thus, larger animals and species closer to humans would likely help translational outcomes. Very few rigorous double blinded clinical trials have been performed to date with standardized ginseng extracts. Large-scale, randomized, double-blinded clinical trials with clear defined outcomes over time are required to delineate the efficacy of ginseng in various neurological disorders in humans.

## Author contributions

CV, Juan Santiago-Moreno and Sylvain Doré were involved in the design of the review. All contributed to performing the literature search, and writing the manuscript. Vaibhav Rastogi and Juan Santiago-Moreno prepared the figures and the table. Sylvain Doré reviewed the figures and table and edited the manuscript.

## Conflict of interest statement

The authors declare that the research was conducted in the absence of any commercial or financial relationships that could be construed as a potential conflict of interest.
